# Health Care Spending Increases and Value in South Korea

**DOI:** 10.1001/jamahealthforum.2024.5145

**Published:** 2025-01-24

**Authors:** Sungchul Park, Joseph L. Dieleman, Marcia R. Weaver, Giryeon Bae, Karen Eggleston

**Affiliations:** 1 Department of Health Management and Policy, Korea University, Seoul, South Korea; 2 Institute for Health Metrics and Evaluation, University of Washington, Seattle; 3 Department of Health Metrics Sciences, University of Washington, Seattle; 4 Department of Global Health, University of Washington, Seattle; 5 Shorenstein Asia-Pacific Research Center, Stanford University, Stanford, California

## Abstract

**Importance:**

Health care spending in South Korea (hereafter Korea) nearly doubled from 2010 to 2019. However, little is known about the drivers and effectiveness of these spending increases in terms of changes in disability-adjusted life-years (DALYs).

**Objectives:**

To evaluate the factors contributing to changes in health care spending and DALYs and estimate the value of health care spending from 2010 to 2019 in Korea.

**Design, Setting, and Participants:**

This cross-sectional study of the population of Korea used 2010 and 2019 data from the National Health Insurance Service for health care spending, and from the Global Burden of Disease 2019 for DALYs. Changes from 2010 to 2019 were decomposed into changes in several factors, including population size, aging, and per-person measures of spending or DALYs. Data analyses were performed from April 2023 to June 2024.

**Main Outcomes and Measures:**

Health care spending was calculated as the total expenditure on medical and long-term care, and health gains were measured as DALYs averted. The value of health care spending was estimated as the ratio of changes in spending per person to changes in DALYs per person.

**Results:**

Total health care spending in Korea increased from $55.0 billion in 2010 to $92.0 billion in 2019. Increases in spending per person accounted for 52.9% of the increase, followed by population aging and population size (35.6% and 11.4%). Total DALYs increased from 11.4 million to 12.2 million. Population aging accounted for 269.4% of the increase, followed by population growth (64.0%). However, DALYs per person decreased (−233.4%), reflecting a lower per capita health burden given the country’s age structure. Assuming 50% and 80% of these health improvements could be attributed to health care spending, the estimated spending per DALY averted was $20 678 and $12 924, respectively. The estimate was slightly larger when excluding DALYs not directly impacted by medical care ($23 687). Korea’s spending per DALY averted is at the lower range of estimates and comparable to that of other high-income countries.

**Conclusions and Relevance:**

This cross-sectional study indicates that increased spending per person, which accounted for half of the total health care spending increase, was associated with improved overall health, evidenced by substantially fewer DALYs. These findings contribute to understanding and evaluating the value of health care spending in Korea.

## Key Points

**Question:**

What is the value of increased health care spending in South Korea (Korea), quantified through disability-adjusted life-years (DALYs)?

**Findings:**

This cross-sectional study of the total population of Korea found that while health care spending increased from $55.0 billion in 2010 to $92.0 billion in 2019 (52.9% per person), DALYs decreased by 233.4% per person. Assuming that at least half of the health improvements were associated with increased spending, the cost per DALY averted was an estimated $20 678 per person.

**Meaning:**

These findings indicate that health care spending in Korea provided a relatively good value from 2010 to 2019, and continuing to quantify its value is essential for assessing the health care system’s performance as it faces a rapidly aging population.

## Teaser

This nationwide cross-sectional study assesses the association of increased health care spending with disability-adjusted life-years in South Korea.

## Introduction

South Korea (hereafter Korea) faces a pressing challenge regarding the sustainability of its health care spending trajectory. In 2010, health care spending in Korea accounted for approximately 6% of the gross domestic product (GDP). Since then, it has surged by nearly 8% per year through 2019.^1^ This growth rate surpasses twice the average annual increase observed among countries in the Organization for Economic Cooperation and Development (3.6%). This upward trend is expected to continue in tandem with the aging population—11.0% of which was composed of older adults (age 65 years and older) in 2010 to 42.5% by 2065—and health care spending is expected to absorb 15.0% of GDP in that year.^2^ These trends raise substantial concerns about the long-term fiscal sustainability of the health care system in Korea.^3,4^

Understanding the drivers underlying the increased spending is crucial for policymakers to pinpoint areas for cost containment.^5,6^ Although some factors—eg, population size and aging—may be beyond the direct control of health policy, others—eg, service prices and utilization rates—may be amenable to targeted policy interventions.^7^ Research in other countries has identified spending per person and spending per case as key drivers of increased spending, reflecting service prices and utilization rates.^8,9,10,11,12^ However, there is limited evidence on the drivers of health care spending in Korea.^13,14^ In Korea, approximately 63.6% of total health care costs were covered by public health insurance in 2010.^15^ To address the financial burden, the government expanded the health insurance benefit package during the past 2 decades, which enhanced access to previously costly health care services and reduced financial burdens associated with Korea’s relatively high out-of-pocket spending.^16,17,18^ However, these expansions may also contribute to increased spending per person as well as continuing differences by income.

Increased spending may potentially translate into improvements in survival and health-related quality of life, and determining whether this spending increase is excessive or justified relies on the magnitude of improvements in health outcomes.^19^ This requires a nuanced approach to estimating the value of the health care system as a whole.^20,21^ However, direct comparisons are challenging because changes in health care spending and health outcomes may be affected by other factors such as population growth and aging, disease incidence and prevalence, and changing health behaviors. Recent developments in methods have made substantial progress in addressing the endogeneity and attribution issues associated with measuring the value of health care spending.^9,21,22^ However, these methods have not been applied to health systems beyond North America and Europe.

This study aimed to evaluate the factors contributing to changes in health care spending and DALYs from 2010 to 2019, and to estimate the value of health care spending and its sensitivity to various assumptions. These analyses were conducted for the entire population as well as by subgroups.

## Methods

This study was reviewed and approved by the institutional review board of the Korea University. Informed consent was waived due to the retrospective nature of the study, in accordance with the applicable guidelines; we adhered to the Strengthening the Reporting of Observational Studies in Epidemiology (STROBE) reporting guideline.

### Data Sources

We analyzed changes in health care spending and health status in nationally representative samples using a cross section of data from January 1 to December 31, 2010, and from January 1 to December 31, 2019. Individual-level data for health care spending was collected from the Korean National Health Insurance Service (NHIS),^23^ and age- and sex-level data for disability-adjusted life-years (DALYs) from the Global Burden of Disease Study 2019 (GBD).^24^ The NHIS data contained claims data from a nationally representative 2% sample of the country’s total population. The national health insurance covers approximately 98% of the total population as the sole public insurer, and its data provide comprehensive information on utilization and spending for inpatient care, outpatient care, prescription drugs, and long-term care.^23^ The GBD aims to quantify the health loss from diseases, injuries, and risk factors at the global and national levels, including estimates for years of life lost (YLLs), years of life lived with disability (YLDs), and DALYs.^24^

Consistent with prior research,^8,9,10,11^ our analysis was conducted at the age and sex levels. While we generally used 5-year age-sex categories, we adopted narrower age categories for children younger than 5 years and a broader category for adults older than 85 years. Thus, we included 19 age categories for each sex, with a total of 38 age-sex categories (eTable 1 in Supplement 1).

### Outcomes

The study’s 2 primary outcome variables were total health care spending and total DALYs. Total health care spending was defined as spending for medical and long-term care. Medical care spending covered inpatient services, outpatient services, and medications; long-term care spending covered home care, residential facility care, and welfare equipment. Spending comprised payments made by the NHIS and cost-sharing payments made by patients. All spending measures were adjusted for inflation using the GDP deflator and expressed in 2021 US dollars. Given that we used the 2% random sample, we multiplied health care spending by 50 to derive estimates representative of the entire population. To comprehensively capture health outcomes, we assessed DALYs averted.

### Factors Contributing to Outcome Changes

Following prior research,^8,9,10,11,12^ we considered 4 distinct factors as drivers of changes in health care spending: population size, age-sex structure (population aging), encounters (ie, visits) per person, and spending per encounter. Given that the GBD estimates DALYs at the population level, data on encounters per person were not available. Thus, our analysis included 3 distinct factors associated with changes in DALYs: population size, age-sex structure, and DALYs per person.

### Statistical Analysis

First, we analyzed the trends in total health care spending (both total spending and spending by service type) and total DALYs (including YLLs and YLDs) from 2010 to 2019. Then, we decomposed these changes into the distinct factors described using a demographic decomposition method originally proposed by Das Gupta in 1993.^25^ This decomposition approach, which has been widely adopted, uses an identity equation to calculate the additive contributions of each factor to changes in health care spending and DALYs.^8,9,10,11,12^ Specifically, we first conducted separate analyses for inpatient care, outpatient care, prescription drugs, and long-term care. Subsequently, estimates from these service types were aggregated to provide a comprehensive perspective on total health care spending. For spending, these analyses were conducted for the entire population and stratified by subgroups for age, sex, health insurance status, disability status, income, and metropolitan residence. For DALYs, the analyses were conducted for the entire population and for the subgroups, age and sex.

Then, we estimated the value of increased health care spending between 2010 and 2019 by calculating the ratio of spending per person (including both encounters per person and spending per encounter) to DALYs per person. Accounting for population growth and aging is assumed to control for changes in the need for health care. This analysis was performed across the entire population and also stratified by age and sex. The value of health care spending was measured in 4 ways. First, we assessed the scenario in which all health improvements were associated solely with increased health care spending. Second, based on studies suggesting that at least half of life expectancy gains were associated with medical advances,^26,27,28^ we assumed that half of the health improvements were associated with increased health care spending,^20^ or equivalently that 2 years of increased spending would be needed to achieve the health gain. Third, given that the rates of deaths from external causes in Korea declined by approximately 20% between 2010 and 2019, a sensitivity analysis assumed that the remaining 80% of the observed health improvements were associated with increased health care spending.^29^ Lastly, we excluded DALYs associated with risk factors that were generally outside of the direct affect of the health care system. Specifically, we calculated total DALYs, subtracting those associated with environmental risks (eg, water, sanitation, and air pollution), behavioral risks (eg, smoking, diet, alcohol use, unsafe sex, and low physical activity), and certain metabolic risks (eg, kidney dysfunction). Considering that conditions such as high blood pressure, high fasting plasma glucose, and high low-density lipoprotein cholesterol can be managed within the health care system, the DALYs associated with these conditions were included in our analysis. Data analyses were performed from April 2023 to June 2024.

## Results

### Study Samples

Both datasets included nationally representative samples of the population of South Korea; however, the original units of analysis differed. For both, older adults were defined as age 65 years and older. The NHIS sample comprised 982 317 individuals in 2010, with 11.7% older adults (n = 115 112; 50.1% females and 49.9% males); and in 2019, it had 929 249 individuals, with 17.8% older adults (n = 165 460; 50.3% females and 49.7% males). The GBD dataset in 2010 comprised 49 554 112 individuals, with 10.8% older adults (n = 5 366 109; 50.2% females and 49.7% males), and in 2019, it totaled 51 764 822 individuals, with 14.8% older adults (n = 7 688 994; 50.1% females and 49.8% males).

### Changes in Health Care Spending

**Figure 1.**
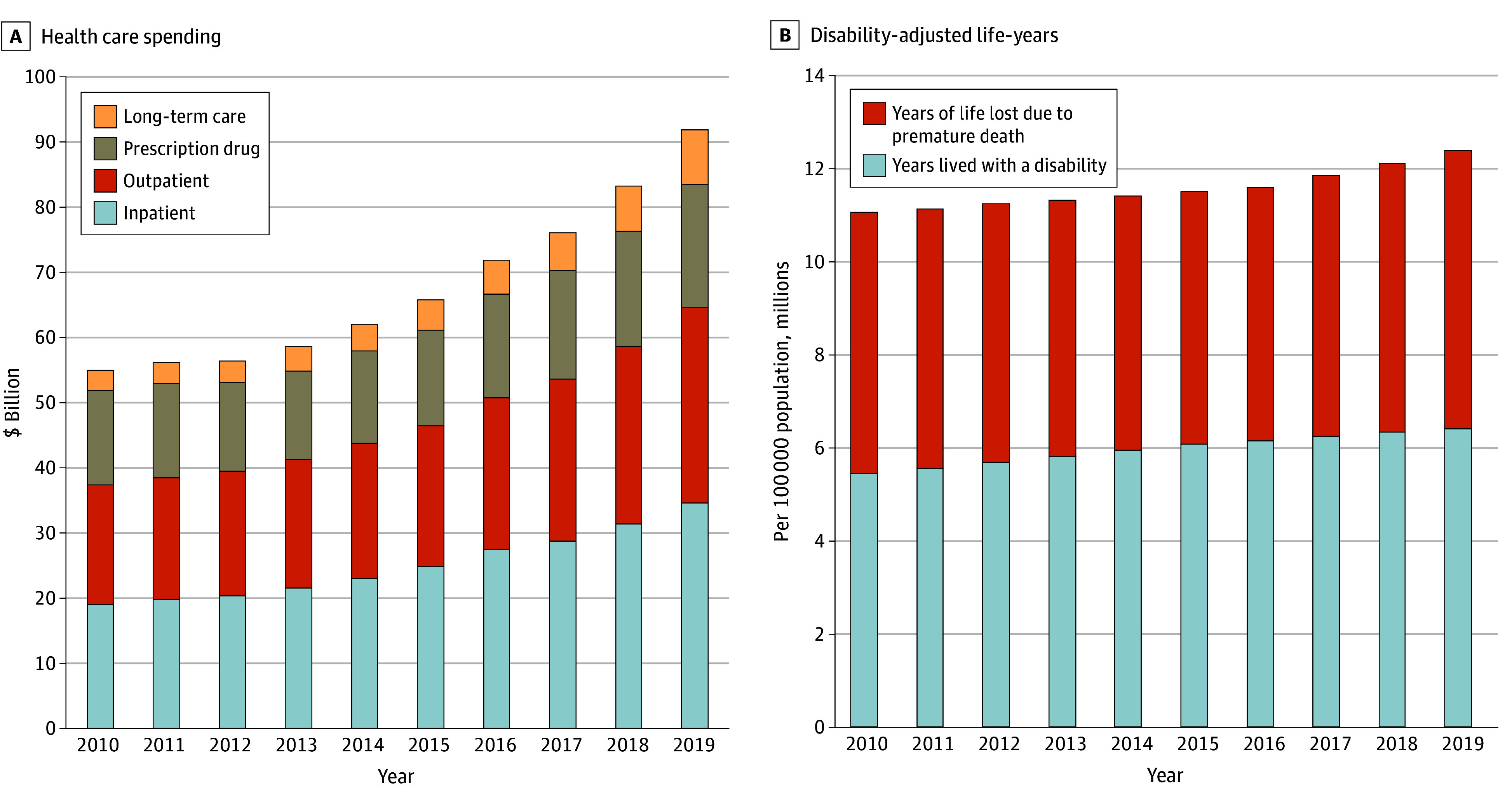
Descriptive Analysis of Health Care Spending and Disability-Adjusted Life-Years in South Korea, 2010 to 2019 Health care spending comprised payments from the National Health Insurance Service^23^ and patients’ out-of-pocket expenses, including costs for inpatient care, outpatient care, prescription drugs, and long-term care. Spending was adjusted for inflation and reported in 2021 US dollars. Inflation adjustments were made using the gross domestic product deflator in South Korea. Disability-adjusted life-years averted were estimated using data from the Global Burden of Disease Study 2019^24^; they comprise years of life lost to premature death and years lived with a disability.

**Figure 2.**
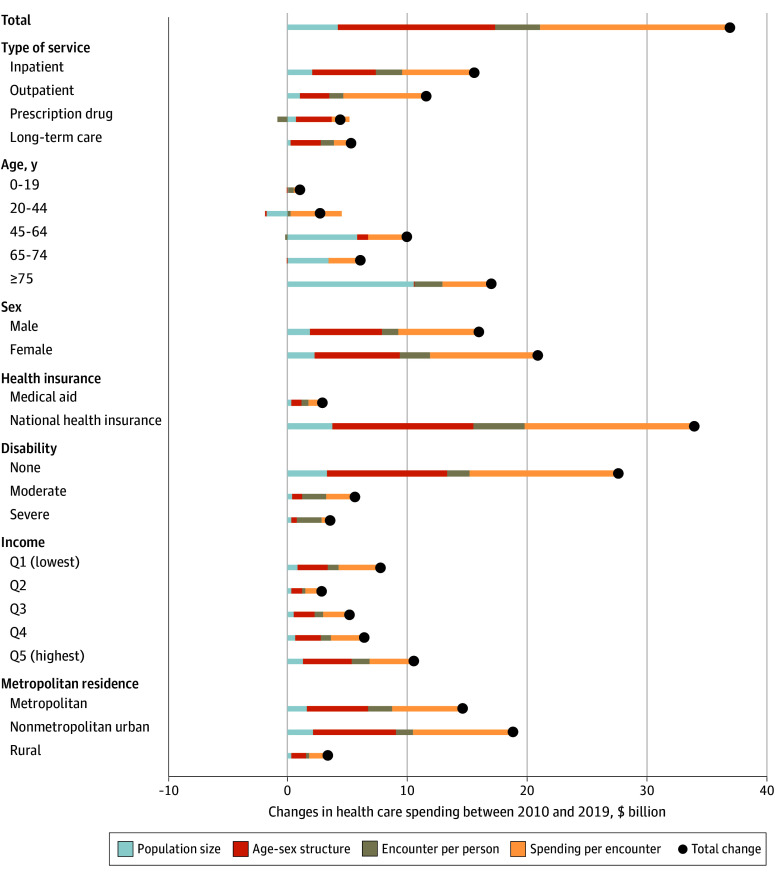
Decomposition Analysis of Changes in Health Care Spending in South Korea, 2010 and 2019 Demographic decomposition method^25^ was used to calculate the additive contributions of each factor to health care spending changes for inpatient care, outpatient care, prescription drugs, and long-term care. These results were aggregated to provide a comprehensive perspective on total health care spending for the entire population and stratified by subgroups. Q indicates quintile.

Descriptive analysis showed that total health care spending in Korea increased from $55.0 billion in 2010 to $92.0 billion in 2019 (Figure 1A), representing an increase in spending per capita from $1211 to $1903, respectively. Korea’s rapidly aging population has often been considered the primary reason for increased health care spending. Although we did find that it plays an important role (accounting for more than $13.2 billion [35.6%]), the most central factor per our decomposition analyses was changes in spending per encounter, which accounted for $15.9 billion (42.9% of the total increase; Figure 2; eTable 2 in Supplement 1). Increases in population size and encounters per person accounted for $4.2 billion (11.4%) and $3.7 billion (10.0%) of the increased spending, respectively.

Across service types, decomposition analyses showed that inpatient spending had the largest increase ($15.6 billion), followed by outpatient spending ($11.6 billion), long-term care spending ($5.4 billion), and prescription drug spending ($4.4 billion; Figure 2; eTable 2 in Supplement 1). While changes in spending per encounter were associated with increased inpatient and outpatient spending (38.4% and 59.5% of the total increase), changes in the age-sex structure were associated with increased prescription drug spending and long-term care spending (67.0% and 46.8%). Notably, a $0.8 billion decrease in prescription drug spending was due to changes in encounters per person, an 18.9% relative decrease that offset the 118.9% increase from other factors.

Although the increases in total health care spending were particularly large among national health insurance enrollees ($34.0 billion) and individuals without disabilities ($27.7 billion), changes in per capita spending were most evident when disaggregating health care spending by income quintile. Specifically, health care spending increased with income, beginning with the second lowest-income group. The highest income quintile had considerably higher spending levels and growth in spending compared to the other quintiles. Notably, the lowest-income quintile, which had the lowest spending in 2010, experienced substantial increases by 2019. Thus, the overall spending level in the lowest-income group was higher than that of the second lowest-income group.

Spending per encounter remained the most important factor, contributing to spending increases across almost all population groups (Figure 2; eTable 2 in Supplement 1), with notable exceptions. First, spending per encounter and encounters per person contributed more to the increases in health care spending among younger age groups, specifically those aged 0 to 19 years (38.5% and 57.8%, respectively) and those aged 22 to 44 years (11.0% and 157.0%, respectively). Changes in population accounted for more of the increase in older age groups. Encounters per person accounted for a larger share of the increase in spending for people with severe disabilities than for spending per encounter.

### Changes in DALYs

**Figure 3.**
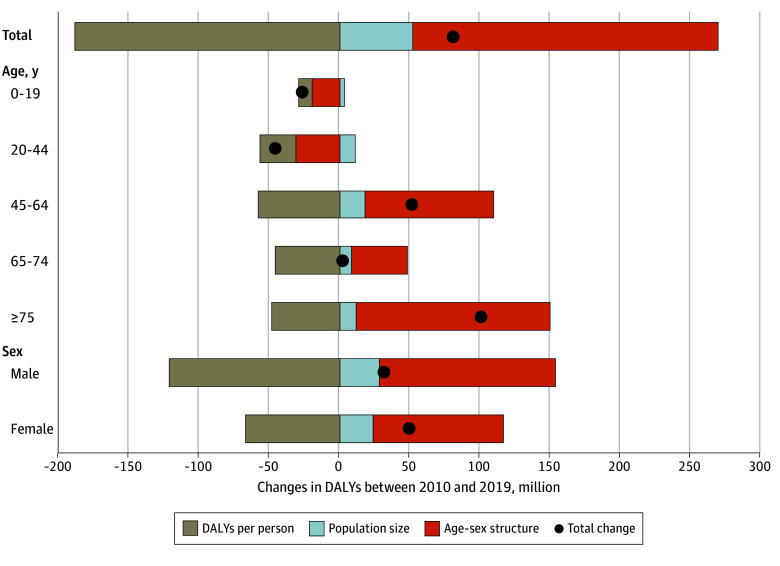
Decomposition Analysis of Changes in Disability-Adjusted Life-years (DALYs) in South Korea, 2010 and 2019 Demographic decomposition method^25^ was used to calculate the additive contributions to changes in DALYs. These analyses were conducted for the entire population and further stratified by subgroups. Due to limited data availability, subgroups were limited to age and sex.

To assess changes in health, we used descriptive analysis to assess the factors underlying the total DALYs increase from 11.4 million in 2010 to 12.2 million in 2019 (Figure 1B). Decomposition analysis demonstrated that the age-sex structure was associated with increases in DALYs (269.4% of the total increase), followed by population growth (64.0% of the total increase; Figure 3; eTable 3 in Supplement 1). However, DALYs per person decreased substantially (−233.4% of the total increase).

Total DALYs decreased among those aged 0 to 19 years and 20 to 24 years, while increasing among those aged 45 to 64 years, 65 to 74 years, and 75 years and older (Figure 3; eTable 3 in Supplement 1). However, DALYs per person decreased across all age groups. DALYs per person decreased more among males than females. After excluding DALYs not directly impacted by the health care system, the decomposition analysis for DALYs showed changes in magnitude; however, the overall trends remained consistent with our original analysis (eTable 4 and eFigure 1 in Supplement 1).

### Value of Health Care Spending

When assuming that all health improvements were associated with increased health care spending, decomposition analysis showed that the estimated spending per DALY averted was $10 339 (Table). When assuming that 50% or 80% of the health improvements could be attributed to increased health care spending, the estimates of spending per DALY averted were $20 678 and $12 924, respectively. The estimate was higher when excluding DALYs not directly impacted by the health care system ($23 687), or when assuming that each DALY averted required 2 ($20 678) or 3 ($31 017) years of medical spending to achieve that health gain.

**Table.** Value of Health Care Spending by Total Population and by Age and Sex in South Korea, 2010 to 2019

Group	Total change in spending, $^a^	Assumptions, health gains due to health care, %						Excluding DALYs beyond health care system control^d^	
		100		80^b^		50^c^			
		Total DALYs change^e^	$/DA^f^	Total DALYs change	$/DA^f^	Total DALYs change	$/DA^f^	Total DALYs change	$/DA^f^
All	19 568 313 261	−1 892 626	10 339	−946 313	20 678	−1 514 101	12 924	−826 105	23 687
Age, y									
0-19	1 040 138 156	−97 307	10 689	−48 654	21 378	−77 845.9	13 361	−43 664	23 821
20-44	4 588 022 619	−257 440	17 822	−128 720	35 643	−205 952	22 277	−112 574	40 756
45-64	3 213 210 327	−583 603	5506	−291 802	11 012	−466 883	6882	−254 259	12 638
65-74	2 667 740 226	−464 484	5743	−232 242	11 487	−371 587	7179	−198 664	13 428
≥75	6 411 983 843	−489 799	13 091	−244 900	26 182	−391 839	16 363	−216 952	29 555
Sex									
Female	11 503 360 368	−672 798	17 098	−336 399	34 196	−538 238	21 372	−307 446	37 416
Male	8 130 337 587	−1 219 828	6665	−609 914	13 330	−975 862	8331	−518 659	15 676

Abbrevations: DA, DALYs averted; DALY, disability-adjusted life-years.

^a^ Estimate derived using decomposed estimates for spending per encounter and encounters per person.

^b^ Assumed that approximately 50% of the observed increase in health outcome was due to health care.

^c^ Rates of death from external causes in Korea declined by approximately 20% between 2010 and 2019. Consequently, we assumed that around 80% of the observed health improvements were associated with increased health care spending.

^d^ DALYs related to environmental/occupational risks, behavioral risks, high body mass index, kidney dysfunction, and low bone mineral density were considered not amenable to health care intervention (ie, beyond the control of the health care system).

^e^ Estimate derived using decomposed estimates for DALYs per person.

^f^ Value of health care spending was estimated as the ratio of changes in spending (2021 US dollars) per person to changes in DALYs per person, reported as dollars per DALY averted.

When assuming half of the health improvements could be attributed to increased health care spending, decomposition analysis demonstrated that individuals aged 20 to 44 years and those aged 75 years and older incurred substantially higher health care spending per DALY averted at $35 643 and $26 182, respectively (Table). However, these estimates were relatively low in the middle-aged groups, with $11 012 for those aged 45 to 65 years and $11 487 for those aged 65 to 74 years. Furthermore, males had lower health care spending per DALY averted compared to females ($13 330 and $34 196, respectively). These estimates remained similar after excluding DALYs not directly impacted by the health care system.

## Discussion

Between 2010 and 2019, per capita health care spending in Korea increased 52.9%, while DALYs per capita decreased 233.4% despite rapid population aging. Assuming half of the health improvements result from increased spending, the estimated spending per DALY averted was $20 678, indicating that health care spending in Korea provides a relatively good value.

The findings of our study, which are consistent with those of prior research,^14^ reveal that the main factor contributing to spending increases was spending per encounter. While there may be multiple explanations for this finding,^7^ the expansion of health insurance is a critical factor. Between 2010 and 2019, Korea underwent 3 phases of health insurance expansion. These expansions aimed to decrease the financial burden of care by reducing cost-sharing, particularly for costly conditions (eg, cancer, cardiac and cerebrovascular diseases). Although some services for these conditions were already covered, those newly added tended to be more costly. Consequently, more individuals gained improved access to previously expensive health care services, alleviating the financial burden of care, which led to more pronounced increases in spending per person rather than a higher number of encounters per person.^16,17,18^ Furthermore, the increase was predominantly due to higher spending per encounter rather than a greater number of encounters per person, suggesting an increase in the delivery of more intensive treatments or costlier technologies and medications.

Prior studies have found that the overall increase in DALYs in Korea has been largely driven by YLDs due to the growing burden of noncommunicable diseases.^30,31^ Our study findings confirm that population aging substantially contributed to the increase in DALYs despite declining DALYs per capita, suggesting improvements in health outcomes during the past decade. Although various factors, including environmental or behavioral risks, may influence the overall DALY burden,^30^ our analysis found that decreases in DALYs per person remained consistent even after excluding for these external risks. This suggests that the observed reduction in DALYs per person may be primarily associated with improvements in diseases and conditions managed by the health care system. This improvement likely reflects advancements in health care delivery, disease management, and public health interventions that address chronic conditions. However, we were unable to investigate the underlying mechanism for this finding, indicating a need for further research to provide more insights.

Korea’s spending per DALY averted is at the lower range of estimates and remains comparable to that of most other high-income countries. Assuming health care spending accounted for half of the health improvements, Korea spent $20 678 per DALY averted between 2010 and 2019. In comparison, a US study reported a median spending of $114 339 per DALY averted between 1996 and 2016.^9^ Adjusting for inflation and methods comparable to our study leaves the estimate virtually identical at $114 489. Our $20 678 estimate for Korea is similar to estimates of spending (all calculated in 2021 US dollars) per quality-adjusted life-years gained in Australia ($20 293)^32^ and Spain ($26 922),^33^ slightly higher than estimates for Canada ($14 249)^34^ and the United Kingdom ($22 193),^35^ and considerably lower than estimates for the Netherlands ($94 719).^36^ Korea’s health care spending growth thus appears to demonstrate reasonable value in terms of improvements in DALYs, although comparisons across studies are challenging due to differences in methods, time periods, and population characteristics. However, these findings warrant cautious interpretation due to relatively lower reimbursement rates under the current payment system. A systematic review of estimates of the value of health care across countries is warranted in the future.

### Limitations

Our study had some limitations worth noting. First, we considered 4 primary drivers for health care spending and DALYs in Korea; however, there may have been additional important factors.^7^ For example, we did not control for changes in disease incidence and prevalence, which raises some concerns about attribution. Second, we observed variations in the factors contributing to changes in health care spending and DALYs, but the precise underlying mechanisms driving these trends remain unclear. Third, our data on total health care spending excludes spending on services not covered by the NHIS, which accounts for approximately 16% of overall health expenditures in Korea (eFigure 2 in Supplement 1). Fourth, the spending data were not disaggregated to specific health conditions; therefore, spending changes could not be compared to health gains by health condition to estimate spending effectiveness or the net value of spending by condition.^9,21^ Fifth, health care spending may affect DALYs over time, with lagged effects particularly for chronic conditions requiring sustained intervention (eg, diabetes). Thus, our findings may underestimate the value of health care spending for this group. Sixth, given the lack of a definitive basis for estimating the extent to which health improvements are driven by medical care, further research is necessary to accurately assess the contribution of medical care to overall health outcomes. Finally, we included estimates from other countries to contextualize our findings, but these results cannot be directly compared due to differences in study methods, time periods, and population characteristics.

## Conclusions

This cross-sectional study found that from 2010 to 2019, increases in spending per person in Korea accounted for half of the total health care spending increase and were associated with a decline in DALYs per capita. Compared to many other high-income countries, spending per DALY averted is at the lower end of estimates, suggesting good value for health care expenditure in Korea. However, since then, the country’s health care spending has surged. Coupled with an aging population, these trends raise substantial concerns about the long-term fiscal sustainability of the health care system. Therefore, it is essential to develop targeted policy interventions for cost containment.

## Notes

Supplement 1. eTable 1 List of 19 age categories for each sex or 38 age-sex categories eTable 2. Decomposition of changes in annual health care spending in South Korea between 2010 and 2019 eTable 3. Decomposition of changes in DALYs in South Korea between 2010 and 2019 eTable 4. Decomposition of changes in DALYs (after excluding DALYs beyond the control of the health care system) in South Korea between 2010 and 2019 eFigure 1. Decomposition of changes in DALYs (after excluding DALYs beyond the control of the health care system) in South Korea between 2010 and 2019 eFigure 2. Percent of health care spending for non-covered services

Supplement 2. Data Sharing Statement
