# Supplementary material for: Health Care Spending Increases and Value in South Korea
Source: JAMA Health Forum. 2025 Jan 24;6(1):e245145. doi: 10.1001/jamahealthforum.2024.5145 (PMC11762226; doi:10.1001/jamahealthforum.2024.5145)
Supplement: Supplement 1. — eTable 1 List of 19 age categories for each sex or 38 age-sex categories eTable 2. Decomposition of changes in annual health care spending in South Korea between 2010 and 2019 eTable 3. Decomposition of changes in DALYs in South Korea between 2010 and 2019 eTable 4. Decomposition of changes in DALYs (after excluding DALYs beyond the control of the health care system) in South Korea between 2010 and 2019 eFigure 1. Decomposition of changes in DALYs (after excluding DALYs beyond the control of the health care system) in South Korea between 2010 and 2019 eFigure 2. Percent of health care spending for non-covered services [file jamahealthforum-e245145-s001.pdf]

# Supplemental Online Content

Park S, Dieleman JL, Weaver MR, Bae G, Eggleston K. Health Care Spending Increases and Value in South Korea. *JAMA Health Forum.* Published online January 24, 2025. doi:10.1001/jamahealthforum.2024.5145

**eTable 1** List of 19 age categories for each sex or 38 age-sex categories

**eTable 2.** Decomposition of changes in annual health care spending in South Korea between 2010 and 2019

**eTable 3.** Decomposition of changes in DALYs in South Korea between 2010 and 2019

**eTable 4.** Decomposition of changes in DALYs (after excluding DALYs beyond the control of the health care system) in South Korea between 2010 and 2019

**eFigure 1.** Decomposition of changes in DALYs (after excluding DALYs beyond the control of the health care system) in South Korea between 2010 and 2019

**eFigure 2.** Percent of health care spending for non-covered services

This supplemental material has been provided by the authors to give readers additional information about their work.

© 2025 Park S et al. *JAMA Health Forum*

eTable 1 List of 19 age categories for each sex or 38 age-sex categories.

| Age-sex category | Age category | Sex category |
|---|---|---|
| 1 | 0 to 11 months | Male |
| 2 | 1 to 4 years | Male |
| 3 | 5 to 9 years | Male |
| 4 | 10 to 14 years | Male |
| 5 | 15 to 19 years | Male |
| 6 | 20 to 24 years | Male |
| 7 | 25 to 29 years | Male |
| 8 | 30 to 34 years | Male |
| 9 | 35 to 39 years | Male |
| 10 | 40 to 44 years | Male |
| 11 | 45 to 49 years | Male |
| 12 | 50 to 54 years | Male |
| 13 | 55 to 59 years | Male |
| 14 | 60 to 64 years | Male |
| 15 | 65 to 69 years | Male |
| 16 | 70 to 74 years | Male |
| 17 | 75 to 79 years | Male |
| 18 | 80 to 84 years | Male |
| 19 | 85 plus years | Male |
| 20 | 0 to 11 months | Female |
| 21 | 1 to 4 years | Female |
| 22 | 5 to 9 years | Female |
| 23 | 10 to 14 years | Female |
| 24 | 15 to 19 years | Female |
| 25 | 20 to 24 years | Female |
| 26 | 25 to 29 years | Female |
| 27 | 30 to 34 years | Female |
| 28 | 35 to 39 years | Female |
| 29 | 40 to 44 years | Female |
| 30 | 45 to 49 years | Female |
| 31 | 50 to 54 years | Female |
| 32 | 55 to 59 years | Female |
| 33 | 60 to 64 years | Female |
| 34 | 65 to 69 years | Female |
| 35 | 70 to 74 years | Female |
| 36 | 75 to 79 years | Female |
| 37 | 80 to 84 years | Female |
| 38 | 85 plus years | Female |

© 2025 Park S et al. *JAMA Health Forum*

eTable 2. Decomposition of changes in annual health care spending in South Korea between 2010 and 2019.

| | Health care spending, $ billion | | | Changes in spending associated with each factor, % | | | |
|---|---|---|---|---|---|---|---|
| | 2010 | 2019 | Change between 2010 and 2019 | Population | Age-sex structure | Encounter per person | Spending per encounter |
| Total | 55.0 | 92.0 | 37.0 | 11.4 | 35.6 | 10.0 | 42.9 |
| Inpatient | 19.0 | 34.6 | 15.6 | 13.5 | 33.8 | 14.3 | 38.4 |
| Outpatient | 18.4 | 30.0 | 11.6 | 9.0 | 21.1 | 10.4 | 59.5 |
| Prescription drug | 14.5 | 18.9 | 4.4 | 17.0 | 67.0 | -18.9 | 34.9 |
| Long-term care | 3.1 | 8.5 | 5.4 | 5.8 | 46.8 | 20.6 | 26.7 |
| Age | | | | | | | |
| 0-19 | 5.9 | 7.0 | 1.1 | 10.1 | -6.4 | 38.5 | 57.8 |
| Inpatient | 1.4 | 2.4 | 1.0 | 13.3 | -3.6 | 34.4 | 55.9 |
| Outpatient | 2.9 | 3.2 | 0.3 | 11.5 | 5.7 | 30.1 | 52.7 |
| Prescription drug | 1.7 | 1.5 | -0.2 | 27.8 | 25.8 | 5.7 | 40.7 |
| Long-term care | 0.0 | 0.0 | 0.0 | 0.0 | 0.0 | 0.0 | 0.0 |
| 20-44 | 10.0 | 12.7 | 2.7 | -62.0 | -6.0 | 11.0 | 157.0 |
| Inpatient | 3.4 | 4.0 | 0.6 | -183.8 | -9.1 | -74.4 | 367.3 |
| Outpatient | 4.3 | 6.4 | 2.1 | -18.1 | -3.8 | 35.0 | 86.8 |
| Prescription drug | 2.3 | 2.3 | 0.0 | -1418.2 | -276.8 | 496.6 | 1298.4 |
| Long-term care | 0.0 | 0.0 | 0.0 | 0.0 | 0.0 | 0.0 | 0.0 |
| 45-64 | 18.2 | 28.2 | 10.0 | 58.4 | 9.5 | -1.6 | 33.7 |
| Inpatient | 6.2 | 10.3 | 4.1 | 62.6 | 6.8 | 11.3 | 19.4 |
| Outpatient | 6.5 | 10.8 | 4.4 | 44.1 | 7.3 | -0.8 | 49.4 |
| Prescription drug | 5.4 | 6.9 | 1.5 | 88.5 | 22.3 | -38.6 | 27.8 |
| Long-term care | 0.1 | 0.1 | 0.0 | 28.3 | 31.7 | 15.4 | 24.6 |
| 65-74 | 10.8 | 16.8 | 6.1 | 56.2 | 0.0 | 0.0 | 43.8 |
| Inpatient | 3.8 | 6.5 | 2.7 | 53.4 | 0.7 | 17.4 | 28.5 |
| Outpatient | 3.1 | 5.3 | 2.2 | 44.8 | -0.4 | -6.7 | 62.3 |
| Prescription drug | 3.2 | 4.1 | 0.9 | 96.1 | -1.2 | -41.1 | 46.2 |
| Long-term care | 0.7 | 1.0 | 0.3 | 39.3 | -0.3 | 27.6 | 33.4 |
| 75+ | 10.2 | 27.2 | 17.0 | 62.0 | 0.4 | 13.7 | 23.9 |
| Inpatient | 4.2 | 11.4 | 7.2 | 62.0 | 1.6 | 13.9 | 22.5 |
| Outpatient | 1.7 | 4.4 | 2.7 | 56.8 | -1.0 | 10.1 | 34.2 |
| Prescription drug | 2.0 | 4.1 | 2.1 | 75.4 | -0.3 | 5.2 | 19.7 |
| Long-term care | 2.3 | 7.4 | 5.1 | 58.9 | -0.4 | 19.1 | 22.3 |
| Sex | | | | | | | |

© 2025 Park S et al. *JAMA Health Forum*

| | | | | | | | |
|---|---|---|---|---|---|---|---|
| Male | 25.0 | 41.0 | 16.0 | 12.0 | 37.3 | 8.3 | 42.3 |
| Inpatient | 9.2 | 16.4 | 7.2 | 14.4 | 36.7 | 6.2 | 42.7 |
| Outpatient | 8.4 | 13.6 | 5.3 | 8.9 | 23.7 | 17.3 | 50.1 |
| Prescription drug | 6.7 | 9.0 | 2.4 | 15.0 | 63.6 | -10.3 | 31.7 |
| Long-term care | 0.8 | 2.0 | 1.2 | 5.3 | 49.0 | 18.4 | 27.2 |
| Female | 30.1 | 51.0 | 20.9 | 11.0 | 34.0 | 11.9 | 43.1 |
| Inpatient | 9.9 | 18.3 | 8.5 | 12.7 | 31.1 | 21.8 | 34.4 |
| Outpatient | 10.1 | 16.4 | 6.3 | 9.2 | 18.9 | 4.7 | 67.3 |
| Prescription drug | 7.8 | 9.9 | 2.1 | 19.2 | 70.8 | -28.5 | 38.5 |
| Long-term care | 2.3 | 6.4 | 4.1 | 6.3 | 44.9 | 22.5 | 26.3 |
| Insurance | | | | | | | |
| National health insurance | 49.2 | 83.2 | 34.0 | 11.2 | 34.6 | 12.5 | 41.7 |
| Inpatient | 16.4 | 30.6 | 14.2 | 13.0 | 32.5 | 20.2 | 34.3 |
| Outpatient | 17.0 | 28.1 | 11.1 | 8.9 | 20.7 | 10.1 | 60.2 |
| Prescription drug | 13.4 | 17.6 | 4.2 | 16.8 | 65.8 | -17.8 | 35.3 |
| Long-term care | 2.4 | 6.9 | 4.5 | 5.8 | 46.5 | 22.4 | 25.3 |
| Medical aid | 5.8 | 8.8 | 3.0 | 11.1 | 29.4 | 19.1 | 40.4 |
| Inpatient | 2.7 | 4.0 | 1.4 | 14.0 | 21.0 | 19.5 | 45.4 |
| Outpatient | 1.4 | 2.0 | 0.6 | 10.1 | 14.5 | 38.0 | 37.4 |
| Prescription drug | 1.1 | 1.3 | 0.2 | 15.0 | 49.6 | -6.6 | 42.1 |
| Long-term care | 0.7 | 1.5 | 0.8 | 6.1 | 48.5 | 11.5 | 33.9 |
| Disability | | | | | | | |
| None | 44.5 | 72.2 | 27.7 | 12.0 | 36.3 | 6.6 | 45.0 |
| Inpatient | 14.8 | 25.3 | 10.5 | 15.6 | 39.2 | 8.7 | 36.4 |
| Outpatient | 15.2 | 25.1 | 9.9 | 8.8 | 19.1 | 8.7 | 63.4 |
| Prescription drug | 12.7 | 16.4 | 3.7 | 16.8 | 66.0 | -18.3 | 35.6 |
| Long-term care | 1.8 | 5.4 | 3.6 | 5.6 | 44.8 | 20.5 | 29.1 |
| Moderate | 4.1 | 9.7 | 5.6 | 6.9 | 15.0 | 36.1 | 42.0 |
| Inpatient | 1.5 | 4.3 | 2.8 | 6.0 | 3.4 | 47.1 | 43.5 |
| Outpatient | 1.1 | 2.1 | 1.0 | 7.9 | 1.5 | 42.3 | 48.4 |
| Prescription drug | 1.1 | 1.7 | 0.6 | 13.7 | 34.2 | -11.7 | 63.9 |
| Long-term care | 0.4 | 1.6 | 1.3 | 5.1 | 41.6 | 29.8 | 23.5 |
| Severe | 6.4 | 10.0 | 3.6 | 10.0 | 12.5 | 57.9 | 19.6 |
| Inpatient | 2.7 | 5.0 | 2.3 | 9.7 | 3.2 | 55.0 | 32.1 |
| Outpatient | 2.0 | 2.8 | 0.8 | 13.5 | 16.5 | 92.6 | -22.6 |
| Prescription drug | 0.7 | 0.7 | 0.0 | 22.8 | 54.2 | 2.3 | 20.8 |

© 2025 Park S et al. *JAMA Health Forum*

| | | | | | | | |
|---|---|---|---|---|---|---|---|
| Long-term care | 1.0 | 1.5 | 0.5 | 6.0 | 47.7 | 21.5 | 24.8 |
| Income | | | | | | | |
| Q1 (lowest) | 7.0 | 14.7 | 7.8 | 10.9 | 33.1 | 11.5 | 44.4 |
| Inpatient | 2.4 | 5.5 | 3.1 | 12.5 | 28.1 | 17.2 | 42.2 |
| Outpatient | 2.3 | 4.7 | 2.3 | 9.2 | 19.5 | 11.0 | 60.3 |
| Prescription drug | 1.8 | 3.0 | 1.1 | 15.7 | 60.5 | -17.3 | 41.1 |
| Long-term care | 0.4 | 1.6 | 1.3 | 5.8 | 46.3 | 24.5 | 23.4 |
| Q2 | 6.9 | 9.7 | 2.9 | 11.1 | 32.7 | 9.1 | 47.0 |
| Inpatient | 2.4 | 3.5 | 1.1 | 14.8 | 37.9 | 5.6 | 41.8 |
| Outpatient | 2.4 | 3.5 | 1.1 | 8.6 | 19.9 | 10.9 | 60.7 |
| Prescription drug | 1.8 | 2.1 | 0.3 | 15.4 | 59.5 | -11.4 | 36.6 |
| Long-term care | 0.3 | 0.6 | 0.4 | 4.4 | 34.6 | 30.4 | 30.5 |
| Q3 | 8.4 | 13.6 | 5.2 | 10.6 | 33.1 | 13.7 | 42.6 |
| Inpatient | 2.8 | 5.1 | 2.3 | 11.8 | 31.6 | 23.7 | 32.9 |
| Outpatient | 3.0 | 4.7 | 1.7 | 8.8 | 20.5 | 9.3 | 61.4 |
| Prescription drug | 2.2 | 2.9 | 0.6 | 15.3 | 59.9 | -15.0 | 39.8 |
| Long-term care | 0.3 | 0.9 | 0.6 | 5.9 | 48.1 | 18.0 | 28.0 |
| Q4 | 11.4 | 17.9 | 6.4 | 10.9 | 33.0 | 12.4 | 43.6 |
| Inpatient | 3.7 | 6.7 | 3.0 | 12.1 | 30.6 | 20.6 | 36.7 |
| Outpatient | 4.1 | 6.2 | 2.2 | 9.0 | 21.3 | 8.9 | 60.9 |
| Prescription drug | 3.2 | 3.8 | 0.7 | 17.1 | 67.1 | -19.7 | 35.4 |
| Long-term care | 0.5 | 1.2 | 0.7 | 5.8 | 46.9 | 20.0 | 27.3 |
| Q5 (highest) | 15.1 | 25.7 | 10.6 | 12.5 | 38.5 | 13.8 | 35.2 |
| Inpatient | 4.8 | 9.2 | 4.4 | 15.4 | 37.7 | 24.1 | 22.8 |
| Outpatient | 5.1 | 8.4 | 3.3 | 9.2 | 21.6 | 10.1 | 59.2 |
| Prescription drug | 4.2 | 5.5 | 1.3 | 19.1 | 75.6 | -23.5 | 28.8 |
| Long-term care | 0.9 | 2.5 | 1.6 | 5.7 | 45.3 | 24.9 | 24.2 |
| Metropolitan residence | | | | | | | |
| Metropolitan | 24.1 | 38.7 | 14.6 | 11.4 | 34.9 | 13.7 | 40.0 |
| Inpatient | 8.1 | 14.5 | 6.4 | 13.4 | 33.9 | 18.3 | 34.4 |
| Outpatient | 8.3 | 13.1 | 4.8 | 8.8 | 20.4 | 13.9 | 57.0 |
| Prescription drug | 6.5 | 8.1 | 1.6 | 17.5 | 69.6 | -18.0 | 30.8 |
| Long-term care | 1.3 | 3.1 | 1.8 | 5.9 | 47.0 | 24.4 | 22.8 |
| Non-metropolitan urban | 24.1 | 43.0 | 18.9 | 11.6 | 36.4 | 7.8 | 44.2 |
| Inpatient | 8.3 | 16.0 | 7.7 | 13.5 | 33.8 | 12.8 | 39.9 |
| Outpatient | 8.1 | 14.1 | 6.0 | 9.2 | 21.5 | 8.3 | 60.9 |

© 2025 Park S et al. *JAMA Health Forum*

| | | | | | | | |
|---|---|---|---|---|---|---|---|
| Prescription drug | 6.3 | 8.9 | 2.6 | 17.1 | 67.8 | -20.9 | 36.1 |
| Long-term care | 1.4 | 4.0 | 2.6 | 5.9 | 47.2 | 19.9 | 27.0 |
| Rural | 6.9 | 10.2 | 3.4 | 10.6 | 35.3 | 9.0 | 45.1 |
| Inpatient | 2.7 | 4.1 | 1.5 | 13.6 | 32.7 | 11.0 | 42.6 |
| Outpatient | 2.0 | 2.7 | 0.7 | 9.3 | 22.8 | 4.4 | 63.4 |
| Prescription drug | 1.7 | 2.0 | 0.3 | 15.4 | 59.3 | -19.3 | 44.7 |
| Long-term care | 0.5 | 1.4 | 0.9 | 5.3 | 41.8 | 18.0 | 34.9 |

© 2025 Park S et al. *JAMA Health Forum*

eTable 3. Decomposition of changes in DALYs in South Korea between 2010 and 2019.

| | Total DALYs | | | Changes in DALYs associated with each factor, % | | |
|---|---|---|---|---|---|---|
| | 2010 | 2019 | Change between 2010 and 2019 | Population | Age-sex structure | DALYs per person |
| Total | 11415479 | 12226459 | 810980 | 64.0 | 269.4 | -233.4 |
| Age | | | | | | |
| 0-19 | 825189 | 557521 | -267668 | -11.2 | 74.9 | 36.4 |
| 20-44 | 2791109 | 2332017 | -459092 | -24.4 | 68.3 | 56.1 |
| 45-64 | 3801173 | 4311866 | 510693 | 34.8 | 179.5 | -114.3 |
| 65-74 | 1935075 | 1953440 | 18365 | 466.0 | 2163.2 | -2529.2 |
| 75+ | 2062933 | 3071615 | 1008682 | 11.2 | 137.3 | -48.6 |
| Sex | | | | | | |
| Male | 6217565 | 6534415 | 316850 | 88.4 | 396.6 | -385.0 |
| Female | 5197914 | 5692044 | 494130 | 48.3 | 187.9 | -136.2 |

© 2025 Park S et al. *JAMA Health Forum*

eTable 4. Decomposition of changes in DALYs (after excluding DALYs beyond the control of the health care system) in South Korea between 2010 and 2019.

| | Total DALYs | | | Changes in DALYs associated with each factor, % | | |
|---|---|---|---|---|---|---|
| | 2010 | 2019 | Change between 2010 and 2019 | Population | Age-sex structure | DALYs per person |
| Total | 10349815 | 11130727 | 780912 | 60 | 251 | -212 |
| Age | | | | | | |
| 0-19 | 776045 | 529568 | -246477 | -12 | 76 | 35 |
| 20-44 | 2565228 | 2155094 | -410134 | -25 | 70 | 55 |
| 45-64 | 3417148 | 3889394 | 472246 | 34 | 174 | -108 |
| 65-74 | 1713872 | 1743238 | 29366 | 259 | 1194 | -1353 |
| 75+ | 1877522 | 2813434 | 935912 | 11 | 135 | -46 |
| Sex | | | | | | |
| Male | 5502025 | 5813109 | 311084 | 80 | 354 | -333 |
| Female | 4847790 | 5317618 | 469828 | 47 | 183 | -131 |

e Figure 1. Decomposition of changes in DALYs (after excluding DALYs beyond the control of the health care system) in South Korea between 2010 and 2019.

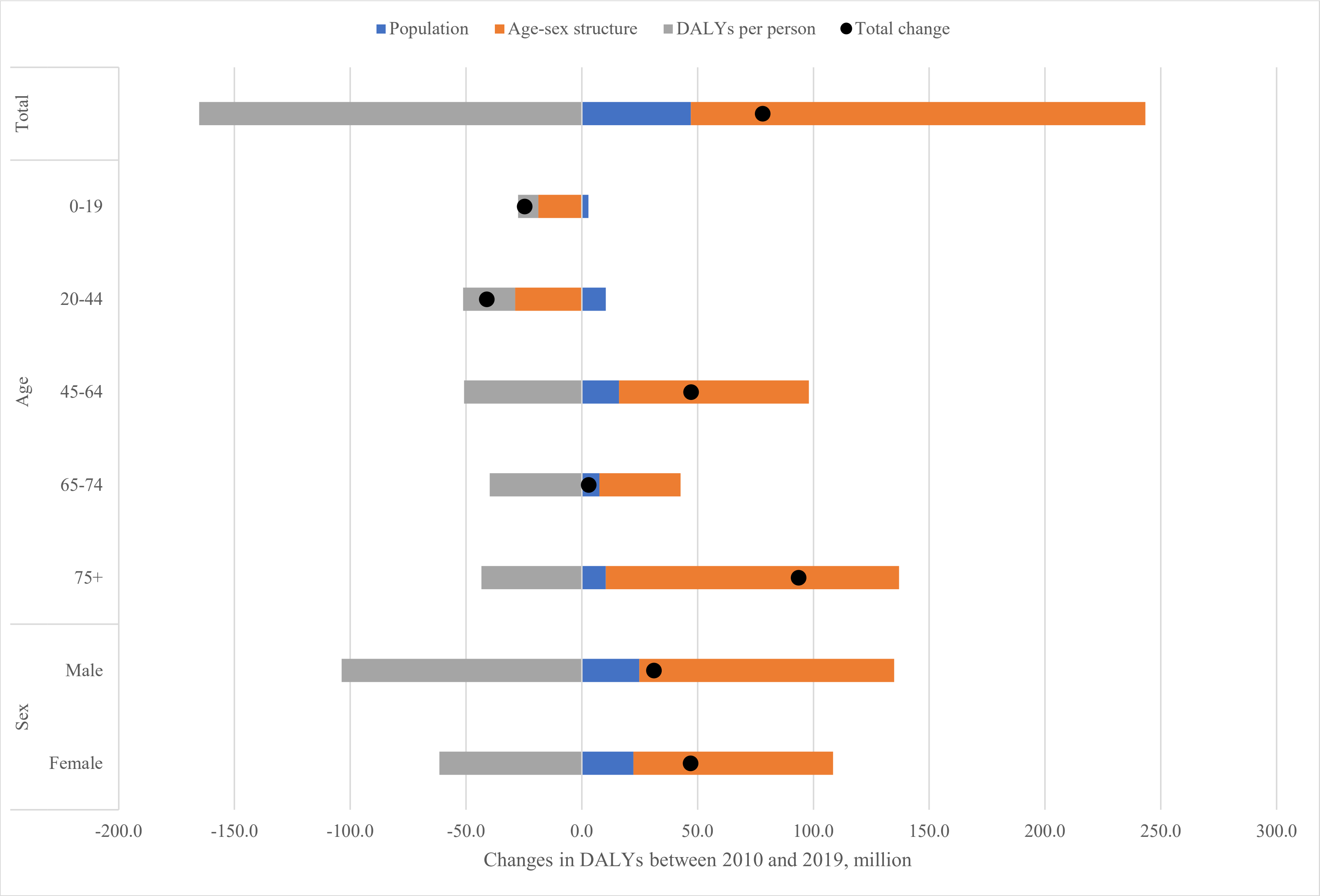


© 2025 Park S et al. *JAMA Health Forum*

e Figure 2. Percent of health care spending for non-covered services

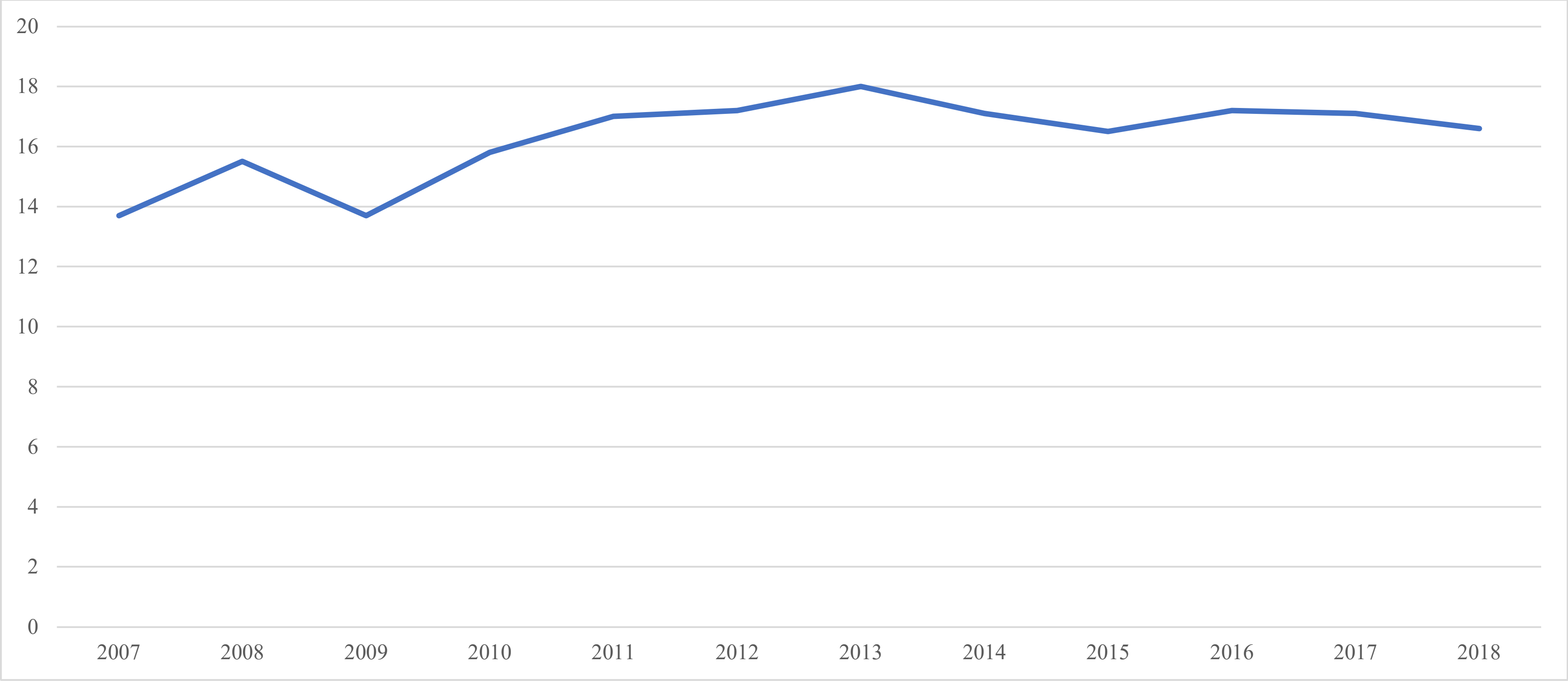


© 2025 Park S et al. *JAMA Health Forum*
