# Supplementary material for: Health Care Spending Increases and Value in South Korea
Source: JAMA Health Forum. 2025 Jan 24;6(1):e245145. doi: 10.1001/jamahealthforum.2024.5145 (PMC11762226; doi:10.1001/jamahealthforum.2024.5145)
Supplement: Supplement 2. — Data Sharing Statement [file jamahealthforum-e245145-s002.pdf]

Data Sharing Statement

Park. Health Care Spending Increases and Value in South Korea. *JAMA Health Forum*. Published January 24, 2025. doi:10.1001/jamahealthforum.2024.5145

Data
Data available: No
